# Impact of polypharmacy phenogroups on different heart failure phenotypes in patients with chronic heart failure: a retrospective examination of real-world cohort

**DOI:** 10.3389/fphar.2025.1526112

**Published:** 2025-05-06

**Authors:** Aseel Sukik, Ahmed Tarek Aboughalia, Abdul Haseeb Said Wali, Amro Al Radaideh, Omar Mohamed Elsayed, Mohammed A. Amer, Joud Said Abuodeh, Oyelola A. Adegboye, AbdelNaser Elzouki, Mohammed Ibn-Mas‘ud Danjuma

**Affiliations:** ^1^ Department of Internal Medicine, Hamad General Hospital, Hamad Medical Corporation, Doha, Qatar; ^2^ College of Medicine, QU Health, Qatar University, Doha, Qatar; ^3^ Department of Internal Medicine, Saint Michael’s Medical Center, Newark, CA, United States; ^4^ Department of Diagnostic Radiology, Hamad Medical Corporation, Doha, Qatar; ^5^ Menzies School of Health Research, Charles Darwin University, Darwin, NT, Australia; ^6^ Weill Cornell College of Medicine, Doha, Qatar; ^7^ NHS Grampian (Dr Grays Hospital), Elgin, Scotland, United Kingdom

**Keywords:** chronic heart failure, polypharmacy, ejection fraction, survival, ICU admissions

## Abstract

**Background:**

Polypharmacy is a rising morbidity amongst patients with chronic heart failure (CHF), with reported prevalence ranging from 70% to 85%. While polypharmacy is essential for managing comorbid conditions, its exact impact on heart failure outcomes is still emerging. This study aims to examine the effects of different polypharmacy phenogroups on mortality and intensive care unit (ICU) admissions across various heart failure phenotypes.

**Methods:**

We conducted a retrospective cross-sectional study involving 4,902 patients with chronic heart failure treated at Hamad Medical Corporation, Doha, Qatar, between January 2018 and January 2022. Patients were classified into three polypharmacy groups: no polypharmacy (0–4 medications), major polypharmacy (five to eight medications), and excessive polypharmacy (≥9 medications). Heart failure phenotypes were categorized based on ejection fraction (EF): reduced EF (HFrEF, <40%), mildly reduced EF (HFmrEF, 40%–49%), and preserved EF (HFpEF, ≥50%). The primary outcome was all-cause mortality, with secondary outcomes including intensive care unit (ICU) admissions.

**Results:**

A cohort of 4,902 patients with chronic heart failure, with a mean age of 61.47 years (SD 15.99), was analyzed. Among them, 51.7% had heart failure with reduced ejection fraction (HFrEF), 16.2% had mildly reduced ejection fraction (HFmrEF), and 32% had preserved ejection fraction (HFpEF). Major polypharmacy due to guideline-directed medical therapy (GDMT), was associated with a significant improvement in survival. In patients with HFpEF, the hazard ratio (HR) for all-cause mortality was 0.62 (95% CI: 0.52-0.75, p < 0.001), while for HFmrEF, it was 0.70 (95% CI: 0.59-0.85, p = 0.001). Conversely, excessive polypharmacy involving non-heart failure medications, was linked to increased ICU admissions (odds ratio [OR]: 1.34, 95% CI: 1.10-1.62, p = 0.02). Cox proportional hazards models demonstrated that excessive polypharmacy was associated with a hazard ratio of 0.11 (95% CI: 0.05-0.23, p < 0.001) for all-cause mortality when the medications were primarily heart failure-specific.

**Conclusion:**

In patients with chronic Heart failure, guideline directed polypharmacy was associated with improved survival, particularly in HFpEF and HFmrEF phenotypes. However, non-heart failure-related polypharmacy is associated with worse outcomes including ICU admissions, necessitating need for targeted interventions for this group of patients.

## Introduction

Polypharmacy in heart failure has recently gotten increasing attention due to the increasing population of both people living with chronic heart failure, as well as the exponential rise in the census of medications required to manage them (Yan et al., 2023a). The prevalence of polypharmacy amongst patients with CHF is variable but has been estimated to exceed 60% (Beezer et al., 2022). In the general population, polypharmacy has been associated with negative outcomes, particularly increased risk of mortality (Leelakanok et al., 2017a). However, in patients with heart failure, this relationship does not appear to be as straightforward and linear as reported in the general population. While polypharmacy is essential for managing the complex needs of heart failure patients, the main concern in these cohorts of patients arises from the potential use of inappropriate medications within these treatment regimens (Prokopidis et al., 2024).

Interestingly, recent evidence challenges the conventional negative view of polypharmacy (Danjuma et al., 2024). A recent cohort study demonstrated that excessive polypharmacy, defined as the use of nine or more medications, including both heart failure-specific and non-heart failure-related medications, was linked to improved survival rates in patients with chronic heart failure (Danjuma et al., 2024). This finding has sparked further interest in understanding the factors contributing to such outcomes, suggesting that polypharmacy in heart failure may require a more nuanced interpretation. In heart failure patients, polypharmacy encompasses various phenogroups, notably guideline-directed medical therapy (GDMT) and medications utilized for the management of other cardiovascular risks (such as antihypertensive medications), both critical components of disease management (Writing Committee Members and ACC/AHA Joint Committee MembersACC/AHA Joint Committee Members, 2022). GDMT includes therapies such as ACE inhibitors (ACE inhibitors), angiotensin receptor blockers (ARBs), mineralocorticoid receptor antagonists (MRAs), angiotensin receptor-neprilysin inhibitors (ARNIs), sodium-glucose cotransporter-2 (SGLT2) inhibitors, and hydralazine/isosorbide dinitrate, all of which have demonstrated substantial mortality benefits, particularly in patients with heart failure with reduced ejection fraction (HFrEF) and mildly reduced ejection fraction (HFmrEF) (Writing Committee Members and ACC/AHA Joint Committee MembersACC/AHA Joint Committee Members, 2022; Burnett et al., 2017; McMurray et al., 2019; Solomon et al., 2022; Packer et al., 2020; Screever et al., 2023; Tran et al., 2018). However, the efficacy of most of these treatments has not been established in heart failure with preserved ejection fraction (HFpEF) patient cohorts. More recently, an increasing number of studies have established survival conferring benefit on HFpEF cohorts exposed to SGLT2 inhibitors, further reinforcing their place in recent heart failure management guidelines (Solomon et al., 2022; Anker et al., 2021). In addition to GDMT, hypertension management plays a critical adjunctive role in the polypharmacy regimens of heart failure patients. Controlling blood pressure is essential for improving outcomes in this population, regardless of their specific heart failure phenotype (Tran et al., 2018; Ziaeian and Fonarow, 2016).

Given these observations, do the different polypharmacy phenogroups—such as GDMT, hypertension-related medications, and non-heart failure medications—have varying impacts on mortality outcomes across the spectrum of heart failure phenotypes? Additionally, considering that heart failure patients often have multiple comorbidities and higher Charlson Comorbidity Index (CCI) scores (Screever et al., 2023; Charlson et al., 2022), are there differences in how these factors, along with demographic characteristics like age and sex, influence outcomes across different heart failure phenotypes and medication phenogroups?

To address these questions, we explored a multi-center multi-ethnic cohort study of patients with chronic heart failure to investigate the prevalence of polypharmacy phenogroups and their association with various heart failure phenotypes, particularly in terms of mortality outcomes.

## Methods

### Study design and population

This was a retrospective study examining a cohort of 4,902 patients with chronic heart failure (CHF) treated at Hamad Medical Corporation, Doha, Qatar. The study spanned from January 2018 to January 2022. Patients were diagnosed based on echocardiographic findings and clinical assessments of heart failure and were followed regularly in specialized heart failure clinics, as well as in other departments addressing their comorbidities. The final adjudication of specific clinico-echocardiographic heart failure group designation for individual patients is left to the treating cardiologist. Inclusion criteria include patients aged ≥18 years with documented heart failure. Exclusion criteria included patients with incomplete medical records or those not on any long-term pharmacotherapy. Patients were included at the time of hospital admission, whether for CLD-related or non-CLD-related reasons. As such, follow-up time began from the point of hospitalization (index admission). We have now revised the Methods section to clearly reflect that patients were enrolled during hospitalization, and not from outpatient clinic visits, to avoid any ambiguity regarding the timing of inclusion and the baseline clinical status of the cohort. The design and the reporting of the study is consistent with the RECORD-PE statement.

### Case Ascertainment

Polypharmacy was defined as the concurrent use of multiple medications, with subcategories based on both the number and type of medications prescribed:

• No polypharmacy: 0–4 medications
• Major general polypharmacy: five to eight medications
• Excessive polypharmacy: ≥9 medications
• Heart Failure-related Polypharmacy: ≥5 medications used for the management of heart failure

The study also distinguished between heart failure-specific polypharmacy, including guideline-directed medical therapy (GDMT), and non-heart failure medications, such as those used for managing comorbid conditions (e.g., hypertension or diabetes).

### Classification of heart failure phenotypes

Heart Failure Phenotypes were classified based on ejection fraction (EF):

• HFrEF (Heart failure with reduced ejection fraction): EF < 40%
• HFmrEF (Heart failure with mildly reduced ejection fraction): EF 40%–49%
• HFpEF (Heart failure with preserved ejection fraction): EF ≥ 50%

Additional covariates included patient demographics (age, sex, nationality), comorbidity burden (assessed using the Charlson Comorbidity Index [CCI]), Primary etiology of heart failure, duration since diagnosis, and length of stay (LOS) during hospital admissions.

### Clinical endpoints

The primary endpoint of the study was all-cause mortality over the study period. Secondary endpoints included length of hospital stay (LOS), intensive care unit (ICU) admissions, and heart failure-specific outcomes related to polypharmacy. These outcomes were stratified by the number of medications taken and the phenotypes of heart failure.

### Data collection and sources

Data was obtained from electronic medical records, capturing detailed medication histories, echocardiographic data, and clinical outcomes. Medications were categorized according to the Multum Lexicon Drug Database, with GDMT drugs including ACE inhibitors, ARBs, MRAs, ARNIs, and SGLT2 inhibitors. Each medication was evaluated for duration of use, and only those taken for at least 4 months were considered to ensure accuracy in polypharmacy classification.

### Statistical analysis

To summarize baseline characteristics of the study cohort, descriptive statistics were used. Continuous variables, such as age and length of stay, were reported as means with standard deviations (SD) or medians with interquartile ranges (IQR), depending on their distribution. Categorical variables, such as sex and comorbidity burden, were presented as frequencies and percentages. Differences between polypharmacy groups (no polypharmacy, major polypharmacy, and excessive polypharmacy) were compared using chi-square tests for categorical variables and Kruskal–Wallis tests for continuous variables. Kaplan-Meier survival analysis was employed to estimate the cumulative probability of survival across different polypharmacy groups and heart failure phenotypes. Differences in survival curves were assessed using log-rank tests. Subgroup analyses were performed to explore survival trends specifically within HFrEF, HFmrEF, and HFpEF cohorts. These analyses aimed to identify whether certain polypharmacy thresholds had varying impacts on mortality depending on the heart failure phenotype. Subsequently multivariable regression models were generated to assess the relationship between polypharmacy and mortality; with adjustment for potential confounders (such as age, sex, CCI and ejection fraction) carried out using Cox proportional hazards regression models. Hazard ratios (HR) and 95% confidence intervals (CIs) were calculated to quantify the effect of polypharmacy levels on survival outcomes. Separate models were run for:

- Total medications (combined heart failure and non-heart failure-related drugs)
- Heart failure-specific medications
- Non-heart failure medications:

A separate generalized additive model (GAM) was fitted to estimate the hazard ratios associated with ejection fraction (EF). GAMs are flexible regression models allowing non-linear relationships between predictors and outcomes by using smoothing splines or other smoothers. The model uses a smoothing function (s (EF)) to model the relationship between EF and hazard ratio non-linearly. To account for potential collinearity between variables, variance inflation factors (VIFs) were calculated, ensuring that all covariates included in the model did not exhibit multicollinearity.

### ICU admission and other outcomes

The association between polypharmacy and ICU admissions was analyzed using binary logistic regression models, with results presented as adjusted odds ratios (aOR) and 95% CIs. Additional exploratory analyses were performed to determine if polypharmacy affected the length of hospital stay, adjusting for similar covariates as in the survival models.

### Sensitivity analysis

We carried out several sensitivity analyses to test the robustness of our findings. These included stratifying the cohort by age groups (<65 years and ≥65 years) and re-analyzing mortality and ICU outcomes to identify age-related effects of polypharmacy. Furthermore, we conducted sensitivity analyses by excluding patients with extreme CCI scores to reduce the potential confounding. All analyses were conducted using Stata version 16. Statistical significance was set at p < 0.05 for all tests, with 95% confidence intervals presented where appropriate.

## Results

### Baseline characteristics

Of the total of 4,902 heart failure patients in this study are summarised in Table 1. The mean age (SD) was 61.47 (15.99) years, and the majority (66%) of the participants were males. Over half of the studied population experienced a reduced EF (HFrEF) level with a prevalence of 51.73% (95% confidence interval (CI): 50.05%, 52.87%). (HFmrEF) is seen in much fewer of the population (16.24%, 95% CI: 15.14%, 17.21%), while a third of the population maintains normal (preserved) EF (HFpEF) levels at 32.03% (95% CI: 30.56%, 33.18%) of the population (Table 1). The HFpEF group also exhibits a higher burden of comorbidities, with the highest mean Charlson Comorbidity Index (CCI) of 4.24 (SD = 2.70) and a greater tendency towards polypharmacy, with 26% of patients in this group on excessive medication regimens. These patients also have more hospital admissions and extended hospital stays, particularly in intensive care units (odds ratio [OR]: 1.34, 95% CI: 1.10-1.62, p = 0.02). Furthermore, the obesity rates differ significantly across the groups, with a substantial portion of HFpEF patients classified as obese.

**TABLE 1:** Patient baseline characteristics and tertiles of ejection fraction.

Characteristics	Overall, N = 4,902	HFrEF: Reduced (≤40), N = 2,536	HFmrEF: Minimal (41-49), N = 796	HFpEF: Preserved (≥50), N = 1,570	p-value^1^
Overall		51.73 (50.05, 52.87)	16.24 (15.14, 17.21)	32.03 (30.56, 33.18)	
Sex, n (%)					<0.001
Male	3,231 (66)	1,992 (79)	543 (68)	695 (44)	
Female	1,673 (34)	544 (21)	253 (32)	875 (56)	
Age, Mean (SD)	61.47 (15.99)	58.33 (15.98)	62.78 (15.51)	65.85 (15.12)	<0.001
Age group, n (%)					<0.001
60–79	40 (0.8)	957 (38)	342 (43)	829 (53)	
40–59	381 (7.8)	1,101 (43)	261 (33)	349 (22)	
80+	1,711 (35)	229 (9.0)	127 (16)	286 (18)	
20–39	2,130 (43)	214 (8.4)	64 (8.0)	103 (6.6)	
<20	642 (13)	35 (1.4)	2 (0.3)	3 (0.2)	
LOS^¥^ days, Mean (SD)	15.54 (43.43)	14.75 (47.86)	15.69 (34.22)	16.75 (39.98)	0.035
No. of admission, Mean (SD)	5.21 (5.87)	4.47 (5.35)	4.97 (5.00)	6.53 (6.80)	<0.001
ICU Stay, n (%)	124 (2.5)	78 (3.1)	15 (1.9)	31 (2.0)	0.041
Deceased, n (%)		552 (22)	168 (21)	392 (25)	0.03
BMI, Mean (SD)		30.04 (18.86)	31.62 (21.74)	33.39 (21.34)	<0.001
Missing		449 (9.2)	138 (5.4)	272 (17.3)	
Obesity, n (%)					<0.001
Pre-obese	1,333 (33)	745 (36)	213 (32)	375 (29)	
Normal	980 (24)	576 (28)	156 (24)	248 (19)	
Obese class 1	783 (19)	386 (18)	145 (22)	252 (19)	
Obese class 3	440 (11)	161 (7.7)	66 (10)	213 (16)	
Obese class 2	408 (10)	162 (7.8)	60 (9.1)	185 (14)	
Underweight	101 (2.5)	57 (2.7)	18 (2.7)	25 (1.9)	
Unknown	859 (17.5)	449 (17.7)	138 (17.3)	272 (17.3)	
CCI, Mean (SD)	3.50 (2.66)	2.98 (2.51)	3.71 (2.64)	4.24 (2.70)	<0.001
CCI group, n (%)					<0.001
Mild	2,001 (41)	1,244 (49)	294 (37)	463 (29)	
Severe	1,254 (26)	649 (26)	286 (36)	712 (45)	
Moderate	1,649 (34)	643 (25)	216 (27)	395 (25)	
Polypharmacy, n (%)					<0.001
No	3,544 (72)	1,967 (78)	549 (69)	1,026 (65)	
Excessive	387 (7.9)	388 (15)	182 (23)	403 (26)	
Major	973 (20)	181 (7.1)	65 (8.2)	141 (9.0)	
Polypharmacy (Heart), n (%)					0.002
No	4,149 (85)	2,188 (86)	653 (82)	1,306 (83)	
Excessive	558 (11)	247 (9.7)	104 (13)	207 (13)	
Major	197 (4.0)	101 (4.0)	39 (4.9)	57 (3.6)	
Polypharmacy (GDMT), n (%)					0.006
No	4,612 (94)	2,357 (93)	756 (95)	1,497 (95)	
Excessive	290 (5.9)	178 (7.0)	40 (5.0)	72 (4.6)	
Major	2 (<0.1)	1 (<0.1)	0 (0)	1 (<0.1)	
Polypharmacy (HTN), n (%)					
No (≤4)	4,637 (95)	2,410 (95)	748 (94)	1,477 (94)	
Excessive	253 (5.2)	121 (4.8)	43 (5.4)	89 (5.7)	
Major	14 (0.3)	5 (0.2)	5 (0.6)	4 (0.3)	
Other polypharmacy, n (%)					<0.001
No	3,892 (79)	2,173 (86)	601 (76)	1,116 (71)	
Excessive	656 (13)	230 (9.1)	121 (15)	305 (19)	
Major	356 (7.3)	133 (5.2)	74 (9.3)	149 (9.5)	

Abbreviation: HFrEF , heart failure with reduced ejection fraction, HFmrEF , heart failure with mildly reduced ejection fraction, HFpEF , heart failure with preserved ejection fraction; LOS , length of stay; ICU , intensive care unit; BMI , body mass index; CCI , charlson comorbidity index; GDMT , Guideline-Directed Medical Therapy; HTN , hypertension.

¥: LOS: Length of in-hospital stay from admission to disposition per admission incident.

When comparing patients’ characteristics based on EF quartiles (Table 2), we observed similar significant associations with EF tertiles (Table 1) for most variables. The first quartile primarily consists of males (81%), but this percentage of males declines steadily in each subsequent quartile, dropping to 41% by the fourth (p < 0.001). Age patterns also show a downward trend, underscoring the older demographic in higher quartiles with significant differences (p < 0.001). The LOS and the number of admissions in the past 5 years increased significantly across the EF quartiles. Additionally, BMI increases significantly with each quartile, and obesity prevalence intensifies, especially in more severe classes. Polypharmacy categories reveal that higher quartiles have more individuals categorised under excessive polypharmacy, highlighting increased medication use.

**TABLE 2:** Patient baseline characteristics and quartiles of ejection fraction.

Characteristic	First quartile, N = 1,395	Second quartile, N = 1,141	Third quartile, N = 1,173	Fourth quartile, N = 1,193	p-value^1^
Sex, n (%)					<0.001
Male	1,130 (81)	862 (76)	747 (64)	491 (41)	
Female	265 (19)	279 (24)	426 (36)	702 (59)	
Age, Mean (SD)	56.72 (16.89)	60.31 (14.55)	63.42 (15.52)	66.20 (14.99)	<0.001
Age group, n (%)					<0.001
60–79	506 (36)	451 (40)	546 (47)	625 (52)	
40–59	611 (44)	490 (43)	346 (29)	264 (22)	
80+	108 (7.7)	121 (11)	186 (16)	227 (19)	
20–39	136 (9.7)	78 (6.8)	93 (7.9)	74 (6.2)	
<20	34 (2.4)	1 (<0.1)	2 (0.2)	3 (0.3)	
Total LOS ([Days], Mean [SD])	15.11 (58.33)	14.31 (30.54)	16.03 (35.96)	16.75 (40.17)	0.031
Number of hospitalizations, Mean (SD)	4.28 (5.09)	4.71 (5.65)	5.43 (5.61)	6.57 (6.85)	<0.001
ICU Stay, n (%)	42 (3.0)	36 (3.2)	30 (2.6)	16 (1.3)	0.019
ICU (Days, Mean (SD)	9.80 (10.47)	8.35 (14.20)	7.55 (8.45)	9.92 (10.46)	0.39
Unknown	1,353	1,105	1,143	1,177	
Died, n (%)	337 (24)	215 (19)	266 (23)	294 (25)	0.003
BMI, Mean (SD)	30.21 (22.90)	29.83 (12.32)	32.11 (20.86)	33.48 (22.08)	<0.001
Unknown	251	198	195	215	
Obesity, n (%)					<0.001
Pre-obese	387 (34)	358 (38)	302 (31)	286 (29)	
Normal	338 (30)	238 (25)	226 (23)	178 (18)	
Obese class 1	207 (18)	179 (19)	195 (20)	202 (21)	
Obese class 3	82 (7.2)	79 (8.4)	118 (12)	161 (16)	
Obese class 2	90 (7.9)	72 (7.6)	111 (11)	134 (14)	
Underweight	40 (3.5)	17 (1.8)	26 (2.7)	17 (1.7)	
Unknown	251	198	195	215	
CCI, Mean (SD)	2.75 (2.48)	3.25 (2.53)	3.84 (2.67)	4.28 (2.69)	<0.001
CCI categories, n (%)					<0.001
Mild	742 (53)	502 (44)	417 (36)	340 (28)	
Severe	322 (23)	327 (29)	452 (39)	546 (46)	
Moderate	331 (24)	312 (27)	304 (26)	307 (26)	
Polypharmacy, n (%)					<0.001
No	1,122 (80)	845 (74)	809 (69)	766 (64)	
Excessive	179 (13)	209 (18)	272 (23)	313 (26)	
Major	94 (6.7)	87 (7.6)	92 (7.8)	114 (9.6)	
Polypharmacy (Heart), n (%)					0.004
No	1,219 (87)	969 (85)	967 (82)	992 (83)	
Major	127 (9.1)	120 (11)	152 (13)	159 (13)	
Excessive	49 (3.5)	52 (4.6)	54 (4.6)	42 (3.5)	
GDMT Category, n (%)					
No	1,293 (93)	1,064 (93)	1,115 (95)	1,138 (95)	
Major	101 (7.2)	77 (6.7)	58 (4.9)	54 (4.5)	
Excessive	1 (<0.1)	0 (0)	0 (0)	1 (<0.1)	
HTN Medications n (%)					
No	1,339 (96)	1,071 (94)	1,101 (94)	1,124 (94)	
Major	54 (3.9)	67 (5.9)	67 (5.7)	65 (5.4)	
Excessive	2 (0.1)	3 (0.3)	5 (0.4)	4 (0.3)	
Other Polypharmacy, n (%)					<0.001
No	1,232 (88)	941 (82)	881 (75)	836 (70)	
Excessive	108 (7.7)	122 (11)	186 (16)	240 (20)	
Major	55 (3.9)	78 (6.8)	106 (9.0)	117 (9.8)	

1Pearson’s Chi-squared test; Kruskal–Wallis rank sum test. LOS: Length of in-hospital stay from admission to disposition per admission incident.

Abbreviation: HFrEF , heart failure with reduced ejection fraction, HFmrEF , heart failure with mildly reduced ejection fraction, HFpEF , heart failure with preserved ejection fraction; LOS , length of stay; ICU , intensive care unit; BMI , body mass index; CCI , charlson comorbidity index; GDMT , Guideline-Directed Medical Therapy (this refers to drugs directed by clinical guidelines for the management of heart failure, HTN , Hypertension; CVS, cardiovascular.

### Comparative mortality outcomes in guideline-directed versus non-guideline-directed polypharmacy

**FIGURE 1:**
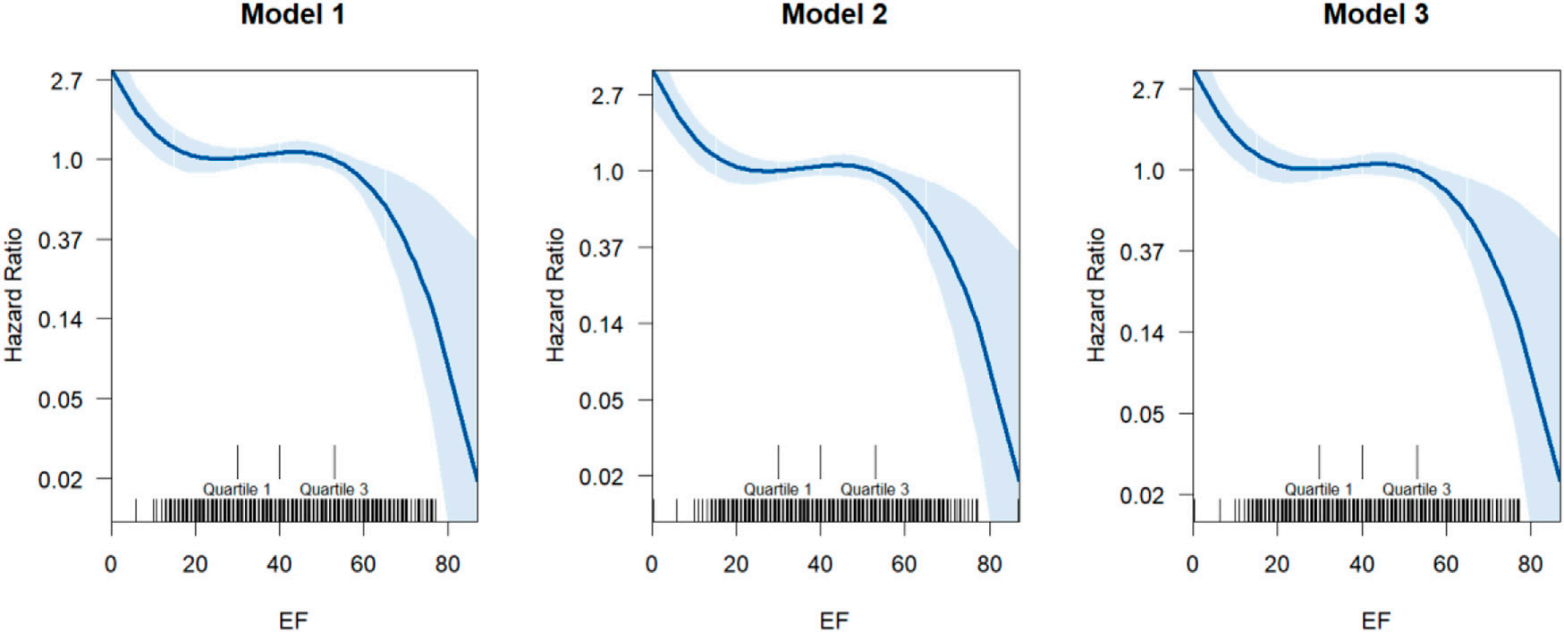
The dose-response relationship between EF and the hazard ratio of incidents of fatal heart failure. The model uses a smoothing function (s (EF)) to model the relationship between EF and hazard ratio non-linearly. These smoothing captures complex relationships without specifying a rigid functional form (linear, polynomial, etc.). The X-axis represents the ejection fraction (EF). The Y-axis shows the hazard ratio (HR), typically modeled using a log-link (log-HR). Solid curves represent the estimated nonlinear effect from the GAM. Shaded areas indicate the confidence intervals around the estimated curve.

Figure 1 presents results from progressively adjusted generalized additive Cox models. Model 1 included ejection fraction. Model 2 is adjusted for ejection fraction, age, and sex, while Model 3 adjusted for ejection fraction, age, sex, CCI and Obesity. All models show a downward trend, indicating the HR for mortality due to heart failure decreases with increased EF with increased uptake of GDMT. The point of inflection occurs around EF value of 40, where the HR starts to level off before decreasing (Supplementary Table S2).

#### All medication

The hazard ratios (HRs) for major and excessive use of medications show significant protective effects against mortality due to heart failure across all models (Supplementary Table S2). For major use, the HRs are consistently low [HR = 0.05 (95% CI: 0.01 - 0.34) in Model 1 and Model 2, HR = 0.07 (95% CI: 0.01, 0.47) in Model 3]. A combination of GDMT for heart failure as well as other drugs for secondary adverse cardiovascular outcomes protection use demonstrates even stronger protective effects [HR = 0.16 (95% CI: 0.08 - 0.31) in Model 1, HR = 0.12 (95% CI: 0.06, 0.24) in Model 2, and HR = 0.11 (95% CI: 0.05, 0.23) in Model 3] across all models. We observed a consistent protective trend across higher EF levels with adjusted models compared to the first EF quartiles. The HR for the second quartile was 0.83 (95% CI: 0.69 - 0.98), and the fourth quartile was 1.11 (95% CI: 0.95 - 1.30), suggesting significant associations between the EF quartile and incidents of fatal heart failure (Supplementary Table S2).

#### Heart medication

Significant reduction in the risks of mortality due to heart failure are observed across all models among patients with excessive polypharmacy (a combination of GDMT for heart failure as well as other CVS protection drugs [HR range from 0.14 to 0.02]). EF showed a similar decrease in risk associated with heart failure related mortality. The protective effect of EF becomes more evident with more complex models. However, the interaction between heart medication polypharmacy and EF was insignificant across the three models.

#### Guideline directed medical therapy (GDMT)

Across Models 1 to 3, major GDMT medication use had a significantly lower risk (HR) of heart failure mortality compared to none, from 0.09 (95% CI: 0.02, 0.37) in Model 1 to 0.12 (95% CI: 0.03, 0.48) in Model 3. Patients in the second to fourth quartile EF had significantly lower HR across the three models than those in the first. For example, in Model 3, patients in the second, third and fourth quartile of EF had a significantly lower HR of 0.70 (95% CI: 0.58, 0.85), 0.69 (95% CI: 0.58, 0.83) and 0.62 (95% CI: 0.52, 0.75), respectively. The interaction between GDMT and EF quartiles does not appear to impact the hazard of mortality due to heart failure incidents significantly (Supplementary Table S2).

#### Hypertension medication

The results for polypharmacy with hypertension medication are similar to GDMT, showing that major use of heart medications is associated with a significantly lower risks of mortality due to heart failure compared to no use and higher EF quartiles are associated with a lower risk of fatal heart failure (Supplementary Table S2).

## Discussion

**FIGURE 2:**
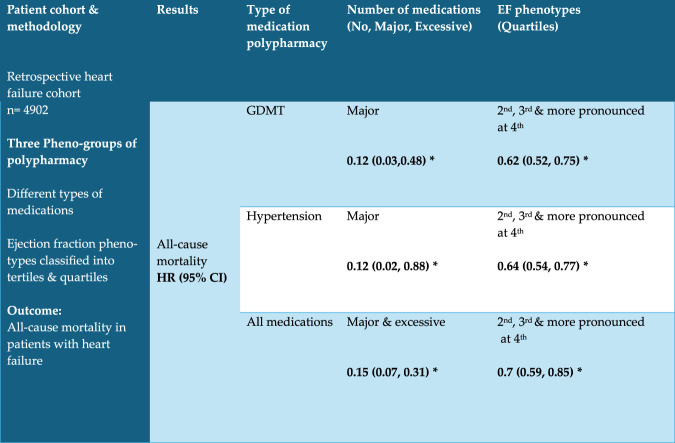
Results are adjusted for polypharmacy, ejection fraction and interaction, age, sex, CCI and Obesity *P < 0.05 Abbreviations: GDMT = Guideline-Directed Medical Therapy.

This study examines the impact of echocardiographic phenogroups on polypharmacy outcomes in patients with chronic heart failure. Our findings (as shown in Figure 2) indicate that guideline-directed medical therapy (GDMT) polypharmacy was associated with a significant reduction in the hazard of fatal heart failure across all models. While higher ejection fractions independently correlated with lower mortality risks, the interaction between EF and polypharmacy levels did not consistently reach statistical significance; this suggests that the mortality benefit of polypharmacy does not significantly vary across EF levels. This aligns with existing literature on the survival benefits of GDMT in chronic heart failure patients (Writing Committee Members and ACC/AHA Joint Committee MembersACC/AHA Joint Committee Members, 2022; Tran et al., 2018). Interestingly, the mortality benefit was most pronounced in patients with an EF of 40% or higher. Despite a higher prevalence of comorbidities and ICU admissions in cohorts with higher EF thresholds (HFmEF and HFpEF), the mortality rate remained comparatively lower. This could be attributed to the superior quality of healthcare typically found in intensive care unit settings. Previous studies have similarly observed improved mortality in HFpEF populations, suggesting that novel treatments such as SGLT2 inhibitors may contribute to these outcomes (Solomon et al., 2022; Packer et al., 2020; Anker et al., 2021). For instance, Steinberg et al. (2012) found that when treated with optimized medical therapy, CHF patients with preserved ejection fraction (HFpEF) had a 50% lower risk of mortality compared to those with reduced ejection fraction. This supports our findings, suggesting that the beneficial effects of GDMT extend across different phenotypes of CHF. This observation is further supported by a 2023 focused update of the 2021 ESC guidelines for the diagnosis and treatment of acute and chronic heart failure. Similarly, McDonagh et al. (2023) and Wang et al. respectively reported a 20% and 30% reduction in mortality among HFpEF patients receiving comprehensive GDMT (McDonagh et al., 2023) (Wang et al., 2024). Similarly, it demonstrated a reduction in mortality in patients with CHF and preserved ejection fraction. This further validates our results, which showed a substantial mortality benefit with GDMT polypharmacy. We also found a higher proportion of ICU admissions amongst patient’s cohorts with non-heart failure related to polypharmacy.

Furthermore, Hypertension-directed polypharmacy was associated with a reduced risks of mortality due to heart failure over a 5-year period. Hypertension-directed polypharmacy showed a similar trend to previously discussed GDMT, particularly in CHF patients with higher EF quartiles (Supplementary Table S1), reinforcing the importance of blood pressure control in achieving better outcomes in HFpEF patients. This is consistent with the already prevailing hypothesis that hypertension contributes significantly to left ventricular hypertrophy and diastolic dysfunction (Moser, 2005b). Ziaeian and Fonarow highlighted that blood pressure control reduces the risk of heart failure-related complications by approximately 25%, which supports findings from our study (Ziaeian and Fonarow, 2016). Our cohort demonstrated that extensive use of all medications was protective against mortality due to heart failure, with this effect being more pronounced at higher ejection fractions. This may be surprising, but it underscores the critical role of GDMT in reducing mortality. The presence of GDMT amongst a “population” of other drugs appears to drive this all-cause mortality benefit. This protective effect of GDMT polypharmacy has been observed in other studies, although contrasting results have been reported in smaller cohorts with shorter follow-up periods. For instance, Danjuma et al. (2024) found a 15% reduction in mortality associated with polypharmacy in chronic heart failure patients, whereas Ozasa et al. observed an increased risk of mortality in a 1-year follow-up study of patients with acute decompensated heart failure (Danjuma et al., 2024; Ozasa et al., 2023). The discrepancies could be attributed to differences in study populations as well as follow-up durations. The benefits observed in our study may be attributed to the basic characteristics of the study cohort; including its comprehensive nature, as well as evidence-based care provided in our institution, with monitored adherence to treatment guidelines and optimal medication management. Until recently patients within this institution had healthcare free at the point of delivery. Matsumoto et al. (2024) reported that adherence to GDMT in HFpEF patients resulted in a 10% reduction in all-cause mortality, highlighting the importance of guideline-directed care (Matsumoto et al., 2024). This supports our observation that polypharmacy, when managed correctly, can lead to significant improvements in patient outcomes.

We also found that female patients comprised the majority of the HFpEF population, which is consistent with previous published reports identifying female sex as a risk factor for HFpEF. Jasinska-Piadlo and Campbell (2023) reported that women were 1.5 times more likely to develop HFpEF compared to male counterparts (Jasinska-Piadlo and Campbell, 2023). Although there are variable reports on this, but factors such as higher obesity rates among women, increased propensity for left ventricular hypertrophy and diastolic dysfunction associated with hypertension, as well as higher life expectancy contribute to this trend. Campbell et al. (2024) noted that women with HFpEF have a 20% higher prevalence of obesity compared to their male counterparts, which aligns with our findings (Campbell et al., 2024).

The Charlson Comorbidity Index (CCI) scores remain one of the most validated surrogates of the effect on comorbidities on clinical outcomes (Charlson et al., 2022). From our study older patients with higher Charlson Comorbidity Index (CCI) scores were more likely to fall into the HFpEF category and be on excessive polypharmacy than age and sex adjusted counterparts. This finding is supported by Shah et al. (2016), who found that HFpEF patients often have a higher burden of comorbidities, with a mean CCI score 1.3 times higher than CHF patients with HFrEF (Shah et al., 2016). We found a clear exponential relationship between higher EF and increased BMI in our study cohort, potentially supporting the observation that obesity is prevalent in more than 80% of HFpEF patients. The underlying hypothesis belying this includes the observation that obesity promotes epicardial adipose tissue expansion and secretion of adipocytokines, leading to inflammation and myocardial fibrosis, ultimately resulting in HFpEF (Packer, 2018). Pandey et al. has described the dose-response relationship for BMI with HFpEF risk, in the form that increasing BMI above the normal range was associated with greater increase in risk of HFpEF which supports our findings (Pandey et al., 2017).

The prevalence of HFrEF in our cohort was consistent with what has thus far been reported in published literature, comprising approximately 50% of total heart failure case burden. Murphy et al. (2020) reported that HFrEF constitutes 40%–50% of heart failure cases globally, which aligns with our findings (Murphy et al., 2020). Additionally, higher CCI scores were observed in patients with HFpEF, supporting the well-documented association between HFpEF and multimorbidity. Sanders-van Wijk et al. (2020) and Paulus and Tschöpe (2013) have both highlighted that patients with HFpEF typically present with a higher number of comorbidities, contributing to the complexity of clinical management evident in these patients' presentation (Sanders-van Wijk et al., 2020; Paulus and Tschöpe, 2013).

Aging populations with increased prevalence of morbidities such as obesity, hypertension, and diabetes also contribute to the higher incidence of HFpEF. Campbell et al. (2024) noted that the prevalence of HFpEF increases significantly with age, particularly in individuals over 65 (Campbell et al., 2024) which is reflected in our cohort. These comorbid conditions are key drivers in the pathophysiology of HFpEF and managing them effectively through polypharmacy can improve patient outcomes. Our study population is comparatively younger (61.47 years) than cohorts typically reported in large multinational heart failure trials (McMurray et al., 2019; Solomon et al., 2022; Tran et al., 2018) where the mean age often ranges between 65 and 75 years. This age difference is a reflection of inevitable demographic, regional, and epidemiologic differences. In the case of Qatar (where our study population was derived from) and the wider Gulf region additional factors such as young migrant populations in these jurisdictions further impacts their age demography. Indeed, multiple epidemiologic studies conducted in the middle east, such as the report from Gulf CARE registry data (Hassan, 2015) and other regional cohorts, have consistently demonstrated that patients with heart failure in this region tend to present at a younger age compared to their Western counterparts. Factors accounting for these includes a higher prevalence of obesity, diabetes, and hypertension at younger ages, as well as a predominantly male expatriate working population, who may have earlier onset of cardiovascular risk factors and less access to preventive care prior to diagnosis and access to healthcare systems.

## Strengths and limitations

One of the significant strengths of our study is its novel investigation into the effects of polypharmacy phenogroups, specifically GDMT, heart failure, and hypertension polypharmacy, across EF spectrum. Our study challenges the common notion that polypharmacy is inherently negative, showing that all medication polypharmacy could have a mortality benefit in patients with CHF; with this effect particularly pronounced at higher ejection fractions thresholds. The robustness of our results is supported by the numerical size of our study cohort, which is a novelty not seen in previous studies. The diverse population in our study, reflective of the demographic composition of Qatar, adds to the generalizability of our findings. Moreover, the patient cohort enrolled into this study adheres to strict guidelines for evidence-based treatment, ensuring that the care provided is of the highest standard. This pharmacy-led strict adherence mechanism likely contributed to the positive outcomes observed in our cohort. Additionally, the detailed analysis of rates and proportions adds depth to our findings and provides a comprehensive understanding of the effects of polypharmacy in different heart failure phenotypes.

Despite its strengths, our study has several limitations that must be acknowledged. The retrospective nature of the study inherently limits the ability to establish causality. Retrospective studies are often subject to biases, such as selection bias and recall bias, which can affect the reliability of the results. The transient follow-up of some patients, particularly seasonal work force, is another limitation. Many of these workforces may have had incomplete follow-up due to returning to their home countries or not renewing medications, which could have impacted on the outcomes observed. This aspect highlights the challenge of ensuring consistent and comprehensive follow-up in a transient population. Additionally, our study focused on all-cause mortality rather than heart failure-specific mortality. While this provides a broad understanding of patient outcomes, it may not capture the nuances specific to heart failure-related deaths. However, it is worth noting that most deaths in chronic heart failure patients were related to heart failure or its complications, which mitigates this limitation to some extent. Another limitation is the potential for unmeasured confounding variables that could influence the results. Although we adjusted for many known confounders, there may be other factors that were not accounted for in our analysis. Finally, the study period did not include recent advances in the management of CHF such as SGLT2i which are now an integral part of CHF management. Future studies with a prospective design and more comprehensive data collection could address these limitations and provide further insights into the effects of polypharmacy on heart failure patients.

## Conclusion

In patients with chronic Heart failure, guideline directed polypharmacy was associated with improved survival, particularly in HFpEF and HFmrEF phenotypes. However, non-heart failure-related polypharmacy is associated with worse outcomes including ICU admissions, necessitating need for targeted interventions for this group of patients.

## Data Availability

The original contributions presented in the study are included in the article/Supplementary Material, further inquiries can be directed to the corresponding author.
