# Supplementary material for: Impact of polypharmacy phenogroups on different heart failure phenotypes in patients with chronic heart failure: a retrospective examination of real-world cohort
Source: Front Pharmacol. 2025 May 6;16:1526112. doi: 10.3389/fphar.2025.1526112 (PMC12088956; doi:10.3389/fphar.2025.1526112)
Supplement: Supplementary file 1 [file Supplementaryfile1.pdf]

Supplementary file.

Supplementary Table S1. Hazard ratios for incidents of mortality due to heart failure with polypharmacy and EF tertiles.

	Model 1		Model 2		Model 3	
**Characteristic**	**HR (95% CI)**1	**p-value**	**HR (95% CI)**1	**p-value**	**HR (95% CI)**1	**p-value**
Overall Medications (All Meds)						
No	—		—		—	
Major	0.00 (0.00 , Inf)	0.98	0.00 (0.00 , Inf)	0.98	0.00 (0.00 , Inf)	0.98
Excessive	0.02 (0.00 , 0.12)	<0.001	0.01 (0.00 , 0.10)	<0.001	0.02 (0.00 , 0.11)	<0.001
EF tertiles						
HFmrEF (41-49)	—		—		—	
HFpEF (≥ 50)	1.15 (0.96 , 1.38)	0.13	0.96 (0.80 , 1.16)	0.7	0.96 (0.79 , 1.18)	0.72
HFrEF (≤ 40)	0.97 (0.81 , 1.15)	0.69	1.15 (0.97 , 1.37)	0.12	1.16 (0.96 , 1.41)	0.12
All Meds* EF tertiles						
Major * HFpEF (≥ 50)	208,067 (0.00 , Inf)	0.98	254,889 (0.00 , Inf)	0.98	139,889 (0.00 , Inf)	0.99
Excessive * HFpEF (≥ 50)	3.12 (0.38 , 25.6)	0.29	3.60 (0.44 , 29.5)	0.23	3.45 (0.42 , 28.3)	0.25
Major * HFrEF (≤ 40)	111,552 (0.00 , Inf)	0.98	101,913 (0.00 , Inf)	0.98	125,307 (0.00 , Inf)	0.99
Excessive * HFrEF (≤ 40)	6.52 (0.84 , 50.5)	0.073	6.82 (0.88 , 52.9)	0.066	5.08 (0.64 , 40.0)	0.12
Heart Medications						
No	—		—		—	
Major	0.04 (0.01 , 0.29)	0.001	0.03 (0.00 , 0.23)	<0.001	0.04 (0.00 , 0.25)	<0.001
Excessive	0.00 (0.00 , Inf)	0.98	0.00 (0.00 , Inf)	0.98	0.00 (0.00 , Inf)	0.98
EF tertiles						
HFmrEF (41-49)	—		—		—	
HFpEF (≥ 50)	1.11 (0.92 , 1.33)	0.27	0.98 (0.81 , 1.18)	0.82	0.95 (0.77 , 1.16)	0.6
HFrEF (≤ 40)	1.04 (0.87 , 1.23)	0.7	1.21 (1.02 , 1.45)	0.029	1.19 (0.98 , 1.44)	0.073
Heart Meds * EF tertiles						
Major * HFpEF (≥ 50)	0.49 (0.03 , 7.88)	0.61	0.57 (0.04 , 9.25)	0.7	0.64 (0.04 , 10.4)	0.76
Excessive * HFpEF (≥ 50)	319,233 (0.00 , Inf)	0.98	350,555 (0.00 , Inf)	0.98	185,077 (0.00 , Inf)	0.98
Major * HFrEF (≤ 40)	1.98 (0.22 , 17.8)	0.54	2.31 (0.26 , 20.8)	0.46	1.90 (0.20 , 18.4)	0.58

Excessive * HFrEF (≤ 40)	159,359 (0.00 , Inf)	0.98	165,452 (0.00 , Inf)	0.98	81,500 (0.00 , Inf)	0.98
GDMT						
No	—		—		—	
Major	0.00 (0.00 , Inf)	0.99	0.00 (0.00 , Inf)	0.99	0.00 (0.00 , Inf)	>0.99
Excessive	0.00 (0.00 , Inf)	>0.99	0.00 (0.00 , Inf)	>0.99	0.00 (0.00 , Inf)	>0.99
EF tertiles						
HFmrEF (41-49)	—		—		—	
HFpEF (≥ 50)	1.15 (0.96 , 1.38)	0.13	1.04 (0.87 , 1.25)	0.65	1.03 (0.84 , 1.25)	0.8
HFrEF (≤ 40)	1.13 (0.95 , 1.34)	0.18	1.34 (1.13 , 1.60)	<0.001	1.32 (1.09 , 1.60)	0.004
GDMT * EF tertiles						
Major * HFpEF (≥ 50)	0.87 (0.00 , Inf)	>0.99	0.82 (0.00 , Inf)	>0.99	0.85 (0.00 , Inf)	>0.99
Excessive * HFpEF (≥ 50)	0.98 (0.00 , Inf)	>0.99	0.65 (0.00 , Inf)	>0.99		
Major * HFrEF (≤ 40)	498,405 (0.00 , Inf)	>0.99	530,983 (0.00 , Inf)	>0.99	694,945 (0.00 , Inf)	>0.99
Excessive * HFrEF (≤ 40)						
Hypertension medications						
No	—		—		—	
Major	0.00 (0.00 , Inf)	0.99	0.00 (0.00 , Inf)	0.99	0.00 (0.00 , Inf)	0.99
Excessive	0.00 (0.00 , Inf)	>0.99	0.00 (0.00 , Inf)	>0.99	0.00 (0.00 , Inf)	>0.99
EF tertiles						
HFmrEF (41-49)	—		—		—	
HFpEF (≥ 50)	1.12 (0.93 , 1.34)	0.23	1.01 (0.84 , 1.21)	0.95	0.99 (0.81 , 1.21)	0.92
HFrEF (≤ 40)	1.07 (0.90 , 1.27)	0.45	1.27 (1.07 , 1.51)	0.007	1.25 (1.04 , 1.52)	0.02
HTN Meds * EF tertiles						
Major * HFpEF (≥ 50)	0.89 (0.00 , Inf)	>0.99	0.96 (0.00 , Inf)	>0.99	1.03 (0.00 , Inf)	>0.99
Excessive * HFpEF (≥ 50)	0.89 (0.00 , Inf)	>0.99	0.84 (0.00 , Inf)	>0.99	1.01 (0.00 , Inf)	>0.99
Major * HFrEF (≤ 40)	420,757 (0.00 , Inf)	0.99	537,932 (0.00 , Inf)	0.99	621,629 (0.00 , Inf)	>0.99
Excessive * HFrEF (≤ 40)	0.93 (0.00 , Inf)	>0.99	0.28 (0.00 , Inf)	>0.99	0.35 (0.00 , Inf)	>0.99
Other medications						
No	—		—		—	
Major	0.00 (0.00 , Inf)	0.98	0.00 (0.00 , Inf)	0.98	0.00 (0.00 , Inf)	0.98
Excessive	0.03 (0.00 , 0.18)	<0.001	0.02 (0.00 , 0.15)	<0.001	0.02 (0.00 , 0.18)	<0.001

EF tertiles						
HFmrEF (41-49)	—		—		—	
HFpEF (≥ 50)	1.17 (0.97 , 1.40)	0.1	0.98 (0.82 , 1.18)	0.86	0.97 (0.79 , 1.19)	0.77
HFrEF (≤ 40)	0.97 (0.81 , 1.15)	0.69	1.16 (0.98 , 1.38)	0.091	1.16 (0.96 , 1.41)	0.12
OtherMeds * EF tertiles						
Major * HFpEF (≥ 50)	97,635 (0.00 , Inf)	0.98	132,223 (0.00 , Inf)	0.98	169,801 (0.00 , Inf)	0.98
Excessive * HFpEF (≥ 50)	2.79 (0.34 , 22.9)	0.34	3.12 (0.38 , 25.6)	0.29	2.91 (0.35 , 23.9)	0.32
Major * HFrEF (≤ 40)	243,911 (0.00 , Inf)	0.98	251,525 (0.00 , Inf)	0.98	179,894 (0.00 , Inf)	0.98
Excessive * HFrEF (≤ 40)	6.76 (0.87 , 52.8)	0.068	6.22 (0.80 , 48.5)	0.081	4.64 (0.59 , 36.7)	0.15

Model 1: Adjusted for polypharmacy, ejection fraction and interaction.
Model 2: Adjusted for polypharmacy, ejection fraction and interaction, age, sex.
Model 3: Adjusted for polypharmacy, ejection fraction and interaction, age, sex, CCI and Obesity.

Supplementary Table S2. Hazard ratios for incidents of mortality due to heart failure with polypharmacy and EF quartiles.

Characteristic	Model 1		Model 2		Model 3	
	**HR (95% CI)1**	**p-value**	**HR (95% CI)1**	**p-value**	**HR (95% CI)1**	**p-value**
All Medications (AllMeds)						
No	—		—		—	
Major	0.05 (0.01 , 0.34)	0.002	0.05 (0.01 , 0.37)	0.003	0.07 (0.01 , 0.47)	0.007
Excessive	0.16 (0.08 , 0.31)	<0.001	0.12 (0.06 , 0.24)	<0.001	0.11 (0.05 , 0.23)	<0.001
EF Quartiles						
First Quartile	—		—		—	
Fourth Quartile	1.11 (0.95 , 1.30)	0.2	0.72 (0.61 , 0.86)	<0.001	0.71 (0.59 , 0.86)	<0.001
Second Quartile	0.83 (0.69 , 0.98)	0.031	0.75 (0.63 , 0.89)	0.001	0.78 (0.64 , 0.94)	0.011
Third Quartile	1.00 (0.85 , 1.17)	0.96	0.77 (0.65 , 0.91)	0.002	0.80 (0.66 , 0.95)	0.014
AllMeds * EF Quartiles						
Major * Fourth Quartile	0.55 (0.03 , 8.91)	0.68	0.60 (0.04 , 9.63)	0.72	0.51 (0.03 , 8.24)	0.64
Excessive * Fourth Quartile	0.35 (0.12 , 0.99)	0.047	0.43 (0.15 , 1.24)	0.12	0.54 (0.18 , 1.59)	0.26
Major * Second Quartile	0.00 (0.00 , Inf)	0.99	0.00 (0.00 , Inf)	0.98	0.00 (0.00 , Inf)	0.99
Excessive * Second Quartile	0.38 (0.10 , 1.41)	0.15	0.48 (0.13 , 1.79)	0.27	0.35 (0.07 , 1.67)	0.19

Major * Third Quartile	0.77 (0.05 , 12.3)	0.85	0.70 (0.04 , 11.3)	0.8	0.00 (0.00 , Inf)	0.99
Excessive * Third Quartile	0.15 (0.03 , 0.71)	0.016	0.17 (0.04 , 0.78)	0.023	0.19 (0.04 , 0.93)	0.04
Heart Medication						
No	—		—		—	
Major	0.14 (0.05 , 0.38)	<0.001	0.13 (0.05 , 0.36)	<0.001	0.12 (0.04 , 0.39)	<0.001
Excessive	0.09 (0.01 , 0.61)	0.014	0.08 (0.01 , 0.60)	0.014	0.10 (0.01 , 0.69)	0.02
EF Quartiles						
First Quartile	—		—		—	
Fourth Quartile	0.95 (0.81 , 1.11)	0.5	0.67 (0.57 , 0.79)	<0.001	0.65 (0.54 , 0.78)	<0.001
Second Quartile	0.78 (0.66 , 0.93)	0.005	0.71 (0.60 , 0.85)	<0.001	0.73 (0.60 , 0.89)	0.001
Third Quartile	0.91 (0.78 , 1.07)	0.28	0.73 (0.62 , 0.86)	<0.001	0.76 (0.64 , 0.91)	0.003
HeartMeds* EF Quartiles						
Major * Fourth Quartile	0.18 (0.02 , 1.59)	0.12	0.18 (0.02 , 1.62)	0.13	0.25 (0.03 , 2.44)	0.23
Excessive * Fourth Quartile	1.32 (0.08 , 21.1)	0.85	1.57 (0.10 , 25.2)	0.75	1.75 (0.11 , 28.1)	0.69
Major * Second Quartile	0.00 (0.00 , Inf)	0.99	0.00 (0.00 , Inf)	0.98	0.00 (0.00 , Inf)	0.99
Excessive * Second Quartile	0.00 (0.00 , Inf)	>0.99	0.00 (0.00 , Inf)	0.99	0.00 (0.00 , Inf)	0.99
Major * Third Quartile	0.20 (0.02 , 1.76)	0.15	0.18 (0.02 , 1.62)	0.13	0.20 (0.02 , 1.92)	0.16
Excessive * Third Quartile	0.00 (0.00 , Inf)	>0.99	0.00 (0.00 , Inf)	0.99	0.00 (0.00 , Inf)	>0.99
GDMT						
No	—		—		—	
Major	0.09 (0.02 , 0.37)	<0.001	0.10 (0.02 , 0.39)	0.001	0.12 (0.03 , 0.48)	0.003
Excessive	0.00 (0.00 , Inf)	>0.99	0.00 (0.00 , Inf)	>0.99	0.00 (0.00 , Inf)	>0.99
EF Quartiles						
First Quartile	—		—		—	
Fourth Quartile	0.88 (0.75 , 1.03)	0.11	0.63 (0.53 , 0.74)	<0.001	0.62 (0.52 , 0.75)	<0.001
Second Quartile	0.74 (0.63 , 0.88)	<0.001	0.69 (0.58 , 0.82)	<0.001	0.70 (0.58 , 0.85)	<0.001
Third Quartile	0.84 (0.71 , 0.99)	0.033	0.67 (0.56 , 0.78)	<0.001	0.69 (0.58 , 0.83)	<0.001
GDMT * EF Quartiles						
Major * Fourth Quartile	0.00 (0.00 , Inf)	0.99	0.00 (0.00 , Inf)	0.99	0.00 (0.00 , Inf)	>0.99

Excessive * Fourth Quartile	1.14 (0.00 , Inf)	>0.99	0.81 (0.00 , Inf)	>0.99		
Major * Second Quartile	0.00 (0.00 , Inf)	0.99	0.00 (0.00 , Inf)	0.99	0.00 (0.00 , Inf)	0.99
Excessive * Second Quartile						
Major * Third Quartile	0.00 (0.00 , Inf)	0.99	0.00 (0.00 , Inf)	0.99	0.00 (0.00 , Inf)	0.99
Excessive * Third Quartile						
HTN Medications						
No	—		—		—	
Major	0.11 (0.01 , 0.76)	0.025	0.12 (0.02 , 0.86)	0.035	0.12 (0.02 , 0.88)	0.037
Excessive	0.00 (0.00 , Inf)	>0.99	0.00 (0.00 , Inf)	>0.99	0.00 (0.00 , Inf)	>0.99
EF Quartiles						
First Quartile	—		—		—	
Fourth Quartile	0.92 (0.78 , 1.07)	0.28	0.65 (0.55 , 0.76)	<0.001	0.64 (0.54 , 0.77)	<0.001
Second Quartile	0.76 (0.64 , 0.91)	0.002	0.70 (0.59 , 0.84)	<0.001	0.71 (0.59 , 0.86)	<0.001
Third Quartile	0.88 (0.75 , 1.04)	0.13	0.70 (0.59 , 0.83)	<0.001	0.72 (0.61 , 0.87)	<0.001
HTNMeds * EF Quartiles						
Major * Fourth Quartile	0.00 (0.00 , Inf)	0.99	0.00 (0.00 , Inf)	0.99	0.00 (0.00 , Inf)	0.99
Excessive * Fourth Quartile	1.10 (0.00 , Inf)	>0.99	2.81 (0.00 , Inf)	>0.99	3.93 (0.00 , Inf)	>0.99
Major * Second Quartile	0.00 (0.00 , Inf)	0.99	0.00 (0.00 , Inf)	0.99	0.00 (0.00 , Inf)	0.99
Excessive * Second Quartile	1.32 (0.00 , Inf)	>0.99	0.94 (0.00 , Inf)	>0.99	2.19 (0.00 , Inf)	>0.99
Major * Third Quartile	0.00 (0.00 , Inf)	0.99	0.00 (0.00 , Inf)	0.99	0.00 (0.00 , Inf)	0.99
Excessive * Third Quartile	1.15 (0.00 , Inf)	>0.99	3.03 (0.00 , Inf)	>0.99	3.41 (0.00 , Inf)	>0.99
Other Medications						
No	—		—		—	
Major	0.08 (0.01 , 0.60)	0.014	0.07 (0.01 , 0.50)	0.008	0.08 (0.01 , 0.55)	0.011
Excessive	0.25 (0.13 , 0.49)	<0.001	0.17 (0.09 , 0.33)	<0.001	0.15 (0.07 , 0.31)	<0.001
EF Quartiles						
First Quartile	—		—		—	
Fourth Quartile	1.12 (0.95 , 1.31)	0.18	0.72 (0.61 , 0.86)	<0.001	0.70 (0.59 , 0.85)	<0.001
Second Quartile	0.82 (0.69 , 0.97)	0.024	0.74 (0.62 , 0.88)	<0.001	0.76 (0.63 , 0.92)	0.005

Third Quartile	1.00 (0.85 , 1.18)	0.97	0.77 (0.65 , 0.90)	0.002	0.79 (0.66 , 0.95)	0.011
OtherMeds* EF Quartiles						
Major * Fourth Quartile	0.34 (0.02 , 5.53)	0.45	0.44 (0.03 , 7.10)	0.56	0.43 (0.03 , 6.96)	0.55
Excessive * Fourth Quartile	0.29 (0.10 , 0.82)	0.02	0.39 (0.14 , 1.13)	0.082	0.52 (0.18 , 1.54)	0.24
Major * Second Quartile	0.53 (0.03 , 8.52)	0.65	0.51 (0.03 , 8.15)	0.63	0.00 (0.00 , Inf)	0.99
Excessive * Second Quartile	0.28 (0.06 , 1.33)	0.11	0.36 (0.08 , 1.68)	0.19	0.40 (0.08 , 1.92)	0.25
Major * Third Quartile	0.00 (0.00 , Inf)	0.98	0.00 (0.00 , Inf)	0.98	0.00 (0.00 , Inf)	0.98
Excessive * Third Quartile	0.14 (0.03 , 0.65)	0.012	0.18 (0.04 , 0.82)	0.027	0.22 (0.05 , 1.06)	0.059

Model 1: Adjusted for polypharmacy, ejection fraction and interaction
Model 2: Adjusted for polypharmacy, ejection fraction and interaction, age, sex
Model 3: Adjusted for polypharmacy, ejection fraction and interaction, age, sex, CCI and Obesity.

Abbreviation: Meds: Medications, EF: Ejection Fraction, HFrEF= Heart Failure with reduced Ejection Fraction, HFmrEF= Heart Failure with mildly reduced Ejection Fraction, HFpEF= Heart Failure with preserved Ejection Fraction, LOS= Length of Stay, ICU= Intensive Care Unit, BMI= Body Mass Index, CCI= Charlson Comorbidity Index, GDMT= Guideline-Directed Medical Therapy, HTN= Hypertension.
